# Introducing an efficient sampling method for national surveys with limited sample sizes: application to a national study to determine quality and cost of healthcare

**DOI:** 10.1186/s12889-021-11441-0

**Published:** 2021-07-17

**Authors:** Mahboubeh Parsaeian, Mahdi Mahdavi, Mojdeh Saadati, Parinaz Mehdipour, Ali Sheidaei, Shahab Khatibzadeh, Farshad Farzadfar, Saeid Shahraz

**Affiliations:** 1 Department of Epidemiology and Biostatistics, School of Public Health, Tehran University of Medical Sciences, Tehran, Iran; 2 National Institute of Health Research (NIHR), Tehran University of Medical Sciences, Tehran, Iran; 3 Harvard T.H. Chan School of Public Health, 677 Huntington Ave, Boston, MA 02115 USA; 4 Department of Computer Science, Iowa State University, Ames, IA 50010 USA; 5 Non-Communicable Diseases Research Center, Endocrinology and Metabolism Population Sciences Institute, Tehran University of Medical Sciences, Tehran, Iran; 6 Heller School for Social Policy and Management, Brandeis University, Waltham, MA USA; 7 Endocrinology and Metabolism Research Center, Endocrinology and Metabolism Clinical Sciences Institute, Tehran University of Medical Sciences, Tehran, Iran; 8 Institute for Clinical Research and Health Policy Studies, Tufts Medical Center, Boston, MA USA

**Keywords:** Survey sampling method, Small sample size, Model-based clustering, Validity, Sampling efficiency, Iran quality of Care in Medicine Program (IQCAMP)

## Abstract

**Background:**

Sampling a small number of participants from an entire country is not straightforward. In this case, researchers reluctantly sample from a single setting or few settings, which limits the generalizability of findings. Therefore, there is a need to design efficient sampling method for small sample size surveys that can produce generalizable results at the country level.

**Methods:**

Data comprised of twenty proxy variables to measure health services demands, structures, and outcomes of 413 districts of Iran. We used two data mining methods (hierarchical clustering method (HCM) and model-based clustering method (MCM)) to create homogenous groups of districts, i.e., strata based on these variables. We compared the internal and stability validity of the methods by statistical indices. An expert group checked the face validity of the methods, particularly regarding the total number of strata and the combination of districts in each stratum. The efficiency of selected method, which is measured by the inverse of variance, was compared with a simple random sampling (SRS) through simulation. The sampling design was tested in a national study in Iran, which aimed to evaluate the quality and costs of medical care for eight selected diseases by only recruiting 300 participants per disease at the country level.

**Results:**

MCM and HCM divided the districts into eight and two clusters, respectively. The measures of internal and stability validity showed that clusters created by MCM were more separated, compact, and stable, thus forming our optimum strata. The probability of death from stroke, chronic obstructive pulmonary disease, and in-hospital mortality rate were the most important indicators that distinguished the eight strata. Based on the simulation results, MCM increased the efficiency of the sampling design up to 1.7 times compared to SRS.

**Conclusions:**

The use of data mining improved the efficiency of sampling up to 1.7 times greater than SRS and markedly reduced the number of strata to eight in the entire country. The proposed sampling design also identified key variables that could be used to classify districts in Iran for sampling from these target populations in the future studies.

**Supplementary Information:**

The online version contains supplementary material available at 10.1186/s12889-021-11441-0.

## Background

### . Problem statement and objectives

Survey is one of the most common instruments to collect population and public health data. National health studies such as ‘STEPwise approach to Surveillance of Non-Communicable Diseases’ (STEPS) [1], ‘Demographic Health Surveys’ (DHS) [2], and health care Utilization studies [3] rely on survey methods. These surveys, to generate generalizable findings, recruit thousands of participants in a multistage sampling design. However, except these national studies, which are priority research for national health authorities, many other surveys particularly in low- and middle-income countries have limited budgets, thereby unable to recruit such a large sample. Practically, they rely on sample sizes that are commonly known as small samples, e.g., less than 500 participants, compared to sample recruited by STEPS or DHS studies. In this case, surveyors usually use a simple random sampling (SRS) or recruit a convenient sample from a single setting or few settings, which reduces generalizability of findings. Therefore, there is a need to design efficient sampling method for small sample size surveys that can produce generalizable results at the country level.

As opposed to SRS, stratified sampling is usually used to increase the efficiency of sampling designs [4, 5]. Stratified sampling classifies a population under study into mutually exclusive subgroups, called strata, and chooses a sample from each stratum. We noticed three main concerns in the use of stratified sampling. Firstly, the strata are usually defined in a convenient manner based on geographical regions such as province [6]. This definition of strata is not always reasonable as obtained strata may not be internally homogenous regarding the outcome of interest. Secondly, stratification based on geographical region considers all regions of a country as strata, while it might be unnecessary to sample from all regions as with an efficient sampling, the number of strata could be fundamentally reduced. This reduction is key to reduce total sample size, which helps make a study more affordable. Thirdly, studies consider only one variable to define strata, while given the complexity of variables that determine health outcomes, multiple variables need to be considered for defining strata [6, 7].

Studies therefore proposed defining strata based on multiple variables to obtain more homogenous definition of strata to improve the efficiency of the stratified sampling. For example, a study in South Korea used prior information of the type of providers (e.g., number of beds and specialized medical units) to define strata of providers, which increased the efficiency of stratified sampling [7]. While their study reduced the number of strata, this reduction was performed based on their judgment rather than letting data or analysis defines the number of strata.

In this research, we proposed a stratified sampling design that uses several proxy variables of health demands, health services structures and health outcomes (DSO) to define homogenous strata of the response variables, instead of the conventional geographical region. The proposed sampling method uses data mining methods to determine the number of strata and the combination of districts in each stratum. We applied this sampling design to a national study called “Iran Quality of Care in Medicine Program” (IQCAMP) for recruiting a small sample of patients for eight selected health condition in Iran.

### Iran quality of care in medicine program

Iran Quality of Care in Medicine Program study aimed to assess the quality of medical care, to examine the elements of the episode of care, and to estimate the overall cost of an episode of care for selected high-cost high-volume diseases in Iran. These diseases are acute myocardial infarction, congestive heart failure, stroke, diabetes mellitus, chronic obstructive pulmonary disease (COPD), major depressive disorder, and end-stage renal disease.

In this study, hundreds of patient-level variables of quality and costs of healthcare were measured at multiple time intervals for 3 months. The size of the sample for the selected conditions is difficult to calculate, because the real effect sizes for the various outcomes are not known. Due to budget constraint, the IQCAMP study could afford to recruit a sample of 300 participants per condition and total of 2400 participants for eight conditions under study. The proposed sampling was applied to this study for recruiting participants in eight surveys with small sample size.

## Methods

We relied on data mining methods for clustering all districts of a country into a minimum number of homogeneous clusters of districts. Data mining includes a wide range of methods, but in this research, we used two types of clustering methods; hierarchical clustering method (HCM) and model-based clustering method (MCM) [8–10] (see section 2.2 for details). The input data to cluster districts consisted of prior information of health demands, health services structures and health outcomes that were available from the national surveys and registries. A key criterion in cluster selection was to minimize the within-cluster differences and at the same time to maximize the between-cluster differences. A cluster of districts forms strata in the stratified sampling.

**Fig. 1:**
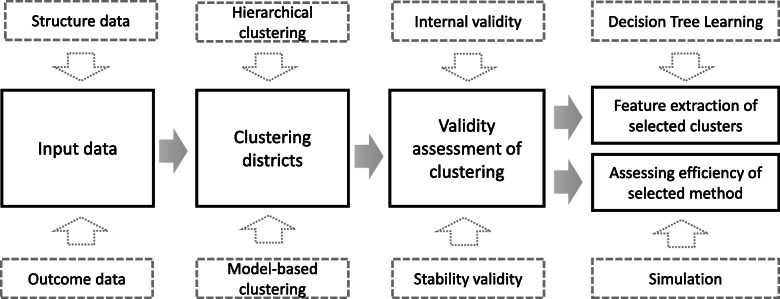
Main steps of the study methods; clustering, validity assessment, and extracting features of clusters Note: An expert panel checked the face validity of all steps

The outline of the method section is as follows: first we explained how we selected DSO indicators and we presented a brief explanation of the clustering methods. Then, we assessed the face validity, internal validity, and stability validity of each clustering methods. We used Decision Tree Learning (DTL) to describe the features of clusters. Subsequently, we conducted a simulation to compare the efficiency of the clustering method with SRS. The schematic diagram of the method is presented in Fig. 1.

### Input data

The input data consisted of micro-level data of DSO indicators. The selected indicators consisted out of patient demands, health services structures, and health outcomes [11, 12]. Patient demands described the characteristics of the population seeking health services, including the type of health needs. Health services structures described insurance arrangements and health care resources that were used to provide services. Health outcomes described the clinical health states of populations. The definition of input variables are presented in Table 1.

**Table 1:** Definition of health demands, health services structures and health outcomes indicators included in the clustering methods

Factor	Variable name	Variable definition	Geographical unit	Data Source	Year
Demand/Disease patterns	Inpatient	Annual average number of inpatient	District	Utilization	2014
	Outpatients	Annual average number of outpatient	District	Utilization	2014
	Hospitalization rate	Hospitalization rate per 1000 population	Province	Hospital Data	2011
	Patient exchange rate	Ratio of sending referrals to receiving referrals (Patient exchange rate)	Province	Hospital Data	2011
	SBP	Mean SBP among hypertensive patients	District	STEPs	2016
	Glucose	Mean of glucose among patients with DM	District	STEPs	2016
	Cholesterol	Mean cholesterol among patients with hyperlipidemia	District	STEPs	2016
Structure	Basic insurance coverage	Basic insurance coverage (% of the population with basic insurance)	District	Utilization	2014
	Complementary insurance coverage	Complementary insurance coverage (% of the population with complementary insurance)	District	Utilization	2014
	Bed density	Number of beds per 1000 population	Province	Hospital Data	2011
	Physician density	Number of physicians per 1000 population	Province	Hospital Data	2011
Outcome	Probability of dying from IHD	Probability of dying from IHD* among adults (age ≥ 30)	Province	DRS	2015
	Probability of dying from Stroke	Probability of dying from Stroke among adults (age ≥ 30)	Province	DRS	2015
	Probability of dying from COPD	Probability of dying from COPD* among adults (age ≥ 30)	Province	DRS	2015
	Probability of dying from Diabetes	Probability of dying from Diabetes mellitus among adults (age ≥ 30)	Province	DRS	2015
	Probability of dying from CKD	Probability of dying from CKD* among adults (age ≥ 30)	Province	DRS	2015
	Neonatal mortality rate	Neonatal mortality per 1000 live births	Province	DRS	2015
	Adverse effect mortality	Mortality rate due to the adverse effect of medical treatment	Province	DRS	2015
	All-cause mortality ratio	Expected mortality rate to observed mortality rate	Province	DRS	2015
	Mortality rate in hospital	Mortality rate among 1000 hospitalized patients	Province	Hospital Data	2011

*SBP* Systolic Blood Pressure, *DM* Diabetes Mellitus, *IHD* Ischemic Heart Disease, *COPD* Chronic Obstructive Pulmonary Disease, *CKD* Chronic Kidney Disease

District is defined as a geographical region with administrative boundaries and an independent network of healthcare provisioning. Province comprises a set of districts and has few managerial authorities in planning and organization of health services. Provincial level is the first level of country subdivisions. Input data consist of data from 31 provinces and 413 districts of Iran

Utilization study measures the use of inpatient and outpatient health services by individuals using a representative sample of population

STEPs is a national survey based on the WHO stepwise approach to study non-communicable disease risk factors

DRS abbreviates Death Registration System

Hospital Data is a research project that studies 0.5% of all inpatient cases in hospitals owned by Ministry of Health and Medical Education in 2011 in Iran

The adverse effect of medical treatment refers to unintended consequences of any types of medical interventions including prevention, diagnosis, treatment, and rehabilitation

We used the data from the national surveys and registries including ‘Iran 2016 STEPwise approach to Surveillance of Non-Communicable Diseases (STEPS) study’ [1], the ‘Death Registration System’ (DRS) in 2015 [13], ‘Iran 2011 Hospital Data’ [14], and ‘Iran 2014 Healthcare Utilization study’ [15]. We used twenty proxy variables to discriminate between different patterns of demands, structures, and outcomes of the diseases under study. Data are aggregated at district or province level, whichever feasible, to create homogeneous clusters. In principle, data are aggregated at district level, which consist of the total 413 districts of Iran. However, in some of the data sources, district level data were unavailable, thus we relied on province level data. The input proxy variables and their aggregation level are shown in Table 1.

### Clustering methods

Before giving the details of the clustering methods, it is necessary to check if data are clusterable, thus it is appropriate to use clustering methods. To check this, we used Hopkins’ statistics, which examines the clustering tendency of the input indicators [16, 17]. The values of Hopkins’ statistic higher than 0.5 were considered clusterable data.

We used two well-known clustering methods: model-based clustering method (MCM) and hierarchical clustering method (HCM) [8–10]. In the model-based clustering, we assume the input data consists of a mixture of probability distributions, each of which represents a different cluster. In this approach, districts with a similar DSO profile are assigned into a same cluster. A best number of clusters and/or cluster distribution are specified based on Bayesian Information Criteria (BIC) [9]. BIC is a criterion for model comparison among a set of models and is partly based on the likelihood function. To reduce overfitting, it introduces a penalty for adding parameters when model fitting. A model with the largest BIC value is considered as an optimum model. We provided the mathematical formulation of the model-based clustering in the Additional File 1-Part A.

Hierarchical clustering method decomposes data hierarchically. The decomposition is undertaken by an agglomerative approach: each observation starts in its own cluster, and pairs of clusters are merged as one moves up the hierarchy [18]. There are different methods for agglomeration of similar observations. We chose the complete method that computes the distance between all objects and merges objects with the least distance. Unlike MCM that can determine the optimum number of clusters, HCM cannot directly estimate this number. We used R package NbClust to estimate an optimum number of clusters for HCM [19]. The package used 30 indices to estimate the optimum number of clusters, i.e., the number recommended by most indices. All statistical analysis is done in R programming language version 3.5.1 and its “mclust” and “stats” packages for MCM and HCM respectively (Additional File 2) [9, 17].

### Validation

#### Face validity of the results of clustering methods

An expert group approved the face validity of the methods. This group consisted of the principal investigator (SSH), Co-investigator (FF), district health networks’ managers, decision makers, and people from healthcare fields. This panel selected input variables, provided insight into the results of the methods, and advised the research team for selecting between methods. The expert panel also consulted with technical teams on the subject matters in selecting input variables and a final clustering method.

#### Comparing internal validity of the clustering methods

Internal validation examines compactness and separation of clusters. Compactness measures within-cluster variations. Separation uses the information of between-cluster variations. Three measures were applied for internal validation: Silhouette width, Dunn index, and within-cluster sum of square [17–19]. Silhouette width compares “the average dissimilarity between a district and other districts within a same cluster” with “average dissimilarity between a district and other districts in other clusters. The values of Silhouette width ranges between − 1 (observation placed in the wrong cluster) and + 1 (observations are well matched to its own cluster). The greater the values of this index, the higher the compactness and separation of the clusters.

Dunn index is calculated as the minimum distance of objects between clusters to the maximum distance of objects in the same cluster. It ranges from zero to infinite. The larger the value of this index, the better the performance of the clustering method. Furthermore, the within-cluster sum of square indicates how closely objects were related in the same clusters. Smaller values of this measure indicated a higher homogeneity of clusters.

#### Comparing stability validation of the clustering methods

We used four indices to measure cluster stability. The measures were the average proportion of non-overlap (APN), the average distance (AD), the average distance between means (ADM), and the figure of merit (FOM) [19]. These measures compare clustering results from the original data with those of data from which one column is removed in a stepwise fashion. For all stability indices, smaller values indicate a better stability.

### Identification of clusters’ features

We used a DTL to describe the clusters’ features in terms of DSO indicators [20, 21]. DTL uses partitioning rules to classify districts into several homogeneous sub-groups based on most important differentiating DSO indicators. Partitioning rule was defined as conditions to assign districts into clusters based on the value of DSO indicators. The algorithm continues the recursive partitioning of data to accurately predict cluster labels.

### Comparison and simulation of sampling methods

We used simulation technique to compare the efficiency of sampling between the clustering method and SRS. Based on the clustering methods, we selected one district per cluster and based on SRS, we selected the same number of districts randomly out of all 413 districts of the country. Next, we estimated the weighted mean of DSO indicators for samples selected using two methods. The weights are proportional to the population of each district to the total population of the selected districts. We simulated these estimates 1000 times and calculated the mean and variance of these estimates. Sampling efficiency was defined by the ratio of the variance of simulated estimates in SRS (${\overline{X}}_{SRS}$) to the variance of simulated estimates in the clustering method (${\overline{X}}_{cluster}$). Larger value for this ratio indicates that the clustering method is more efficient than the SRS method.

## Results

**Fig. 2:**
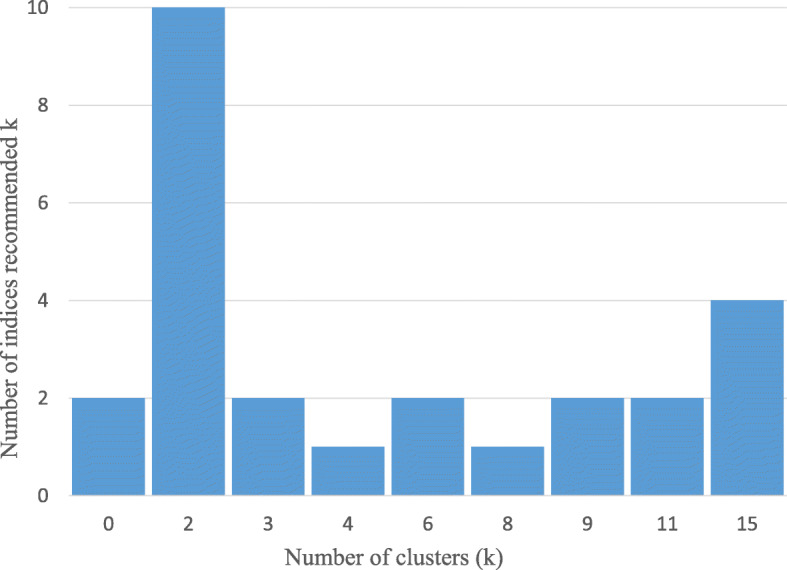
Proposed number of clusters by NbClust package

The Hopkins’ statistic of input measures was estimated as 0.67, indicating a good clustering tendency. Figure 2 demonstrates the number of clusters recommended by different statistical indices. The X-axis shows the number of recommended clusters (k) and the Y-axis shows the number of indices proposed k. Most indices recommended two clusters, which was considered as the optimum number of clusters in HCM. Whereas MCM recommended eight clusters based on the BIC criteria (Additional File 1-Part B).

### The validity of clustering methods

We compared the internal validity of results from MCM and HCM. We added another scenario to test the performance of HCM with eight clusters (HCM-8) (Table 2). The within-cluster sum of square in MCM with eight clusters (MCM-8) was lower than HCM with two clusters (HCM-2). The Dunn index of MCM-8 was higher than that of HCM-2. These results indicate that MCM-8 clusters are more compact and separated than HCM-2. However, the average silhouette width of HCM-2 is larger than MCM-8. Comparing the clustering methods with the same number of clusters, the average silhouette width of MCM-8 is larger than HCM-8. Thus, the model-based method outweighs the hierarchical method with a same number of clusters.

**Table 2:** Comparison of internal and stability validity by clustering methods

Validity Indexes	MCM-8 Model-based with eight clusters	HCM-2 Hierarchical with two clusters	HCM-8 Hierarchical with eight clusters (Additional scenario)
Internal Validity Indexes			
Within-clusters Sum of Squares^a^	**255.87**	384.55	292.65
Average silhouette width^b^	0.14	**0.17**	0.09
Dunn index^b^	**0.27**	0.20	0.19
Stability Validity Indexes			
Average Proportion of Non-overlap (APN)^c^	0.13	**0.09**	0.19
Average Distance AD^c^	**1.11**	1.37	1.24
Average distance between means (ADM)^c^	**0.19**	0.19	0.28
Figure of Merit (FOM)^c^	**0.21**	0.24	0.22

^a^ The lower the value of the within-cluster sum of square, the higher the extent of compactness

^b^ The higher the value of Dunn index and average silhouette width, the higher the extent of compactness and separation

^c^ For all stability indices, smaller values indicate better stability validity

**Fig. 3:**
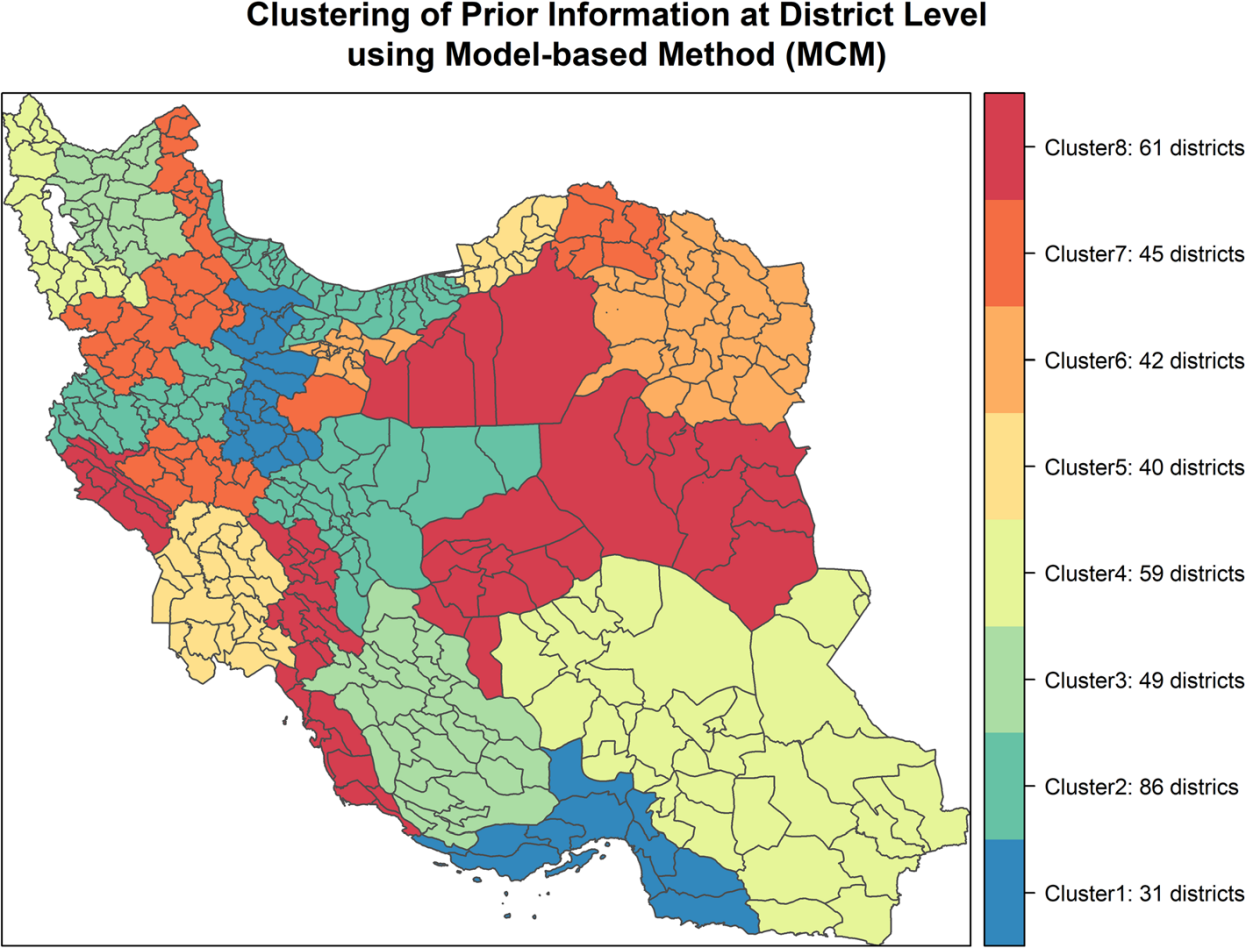
Geographic distribution of 8 clusters identified by the Model-based Clustering Method

The results of four stability measures are given in Table 2. AD, ADM and FOD selected MCM-8 as a more stable model, whereas APN identified HCM-2 as a more stable model. Based on internal and stability validity, we selected MCM with eight clusters as a final classification of districts in this study. The geographic distribution of clusters and the districts of each cluster in MCM-8 is depicted in Fig. 3.

The number of districts in clusters varies from 31 to 86. Cluster 1 has the least number of districts and cluster 2 has the largest. Since the input data is at the district level, MCM assigns districts into clusters. To generalize the clustering result to province level, we assigned a province to a cluster that the majority of districts and the largest weighted population of that province fall into that cluster. To select one province per cluster, we calculated the distance of each province from other provinces in the same cluster and selected the province with minimum distance from other provinces (Additional File 1-Part C).

### Features of identified clusters

**Fig. 4:**
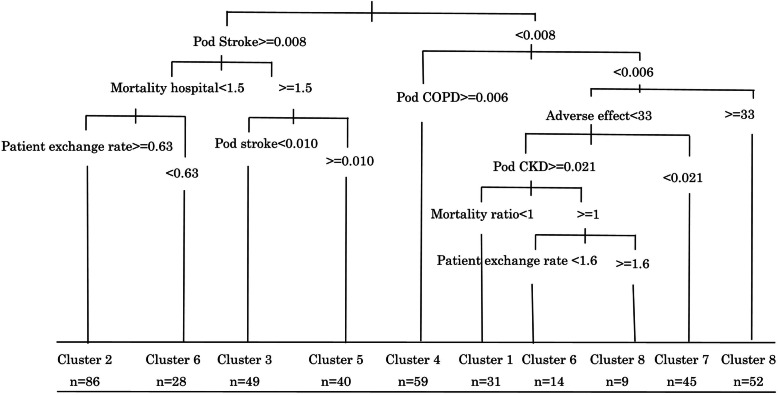
Using decision tree learning to describe distinctive features of 8 clusters identified by the Model-based Clustering Method

The features of MCM-8 clusters are shown in Fig. 4. The most significant DSO indicators that make distinctions between clusters were the probability of death from stroke, the probability of death from COPD, in-hospital mortality rate, patient’s exchange rate, the mortality rate caused by the adverse events of medical treatment, the probability of death from Chronic Kidney Disease (CKD), and all-cause mortality ratio (Fig. 4).

The decision tree identified 10 partitioning rules. Except for clusters six and eight, other clusters had unique features and were identified by only one partitioning rule. For instance, the distinct features of cluster 1 were as follows: all 31 districts had the probability of death from stroke < 0.008, the probability of death from COPD < 0.006, the mortality by adverse events of medical treatment < 33, the probability of death from CKD ≥0.021, and the all-cause mortality ratio < 1. These values were considered as cut-off points for partitioning. DTL accurately placed all 31 districts in this cluster.

Per cluster eight and six, DTL identified two rules. In cluster eight, out of 61 districts, 52 were identified by one rule and nine districts by another. These rules were similar in the probability of death from stroke and the probability of death from COPD while they were different in the mortality rate caused by the adverse events of medical treatment, the probability of death from CKD, the all-cause mortality ratio, and the patient exchange rate. Similarly, among 42 districts in cluster six, 28 districts had one partitioning rule and 14 districts were identified by the other partitioning rule (see cluster’s features in Fig. 4).

### Assessing the efficiency of clustering method

Table 3 illustrates the sampling efficiency of key features of MCM-8 clusters detected by the DTL. The simulation results showed that the clustering method decreased the sampling variance of all these features compared to SRS. The highest reduction in a sampling variance, by 1.7 times, was related to the probability of death from stroke. The next higher reduction, 1.5 times, was for the probability of death from COPD and the patient exchange rate. The lowest reduction was related to the mortality rate attributed to the adverse events of medical treatments with sampling efficiency 1.2.

**Table 3:** Comparison of efficiency of clustering-based sampling to SRS based on distinct cluster features

Variables	Sampling efficiency $\frac{{\mathbf{Variance}}_{\boldsymbol{SRS}}\left(\overline{\boldsymbol{X}}\right)}{{\mathbf{Variance}}_{\boldsymbol{Clustering}}\left(\overline{\boldsymbol{X}}\right)}$
The ratio of sending referrals to receiving referrals (Patient exchange rate)	1.5
The probability of dying from Stroke (age ≥ 30)	1.7
The probability of dying from COPD^a^ (age ≥ 30)	1.5
The probability of dying from CKD^b^ (age ≥ 30)	1.4
The mortality rate attributed to the adverse effect of medical treatment	1.2
Expected mortality rate to observed mortality rate	1.3
The mortality rate among 1000 hospitalized patients	1.4

^a^ COPD: Chronic Obstructive Pulmonary Disease

^b^ CKD: Chronic Kidney Disease

## Discussion

We used a data mining method to satisfy the sampling design requirements of the IQCAMP, a national pilot survey with a limited budget and sample size. The model-based clustering method divided districts into eight clusters, whereas the hierarchical clustering method divided districts into two clusters. Before conducting the validity assessment through statistical analysis an expert group approved the face validity of the methods. The internal validity as measured by the within-cluster sum of square and Dunn index showed that the clusters of districts in MCM-8 had higher compactness and separation in comparison with HCM-2. Moreover, most stability indices recognized that MCM with eight clusters is more stable than HCM with two clusters. Therefore, we selected MCM with eight clusters as the final strata in the stratified sampling design. These clusters were mainly characterized by the probability of death from stroke, COPD, and CKD, in-hospital mortality rate, patient’s exchange rate, the mortality rate attributed to adverse events of medical treatment, and all-cause mortality ratio.

In the use of clustering methods, we built on earlier studies [7, 22]. Though there exist many clustering methods, we used the MCM, which has also been extensively used in the literature [23, 24]. The main advantages are that it relies on statistical models and requires no pre-specified number of clusters [25].

Our proposed method could be discussed in the light of representativeness and efficiency of the sample estimates. Regarding the representativeness, we clustered the country into homogeneous groups of districts and selected one district per group. Thus, our sample was systematically selected from all of them. We do not claim representativeness in a statistical sense for sample estimates; yet the representativeness is inherently present in the procedures that are taken by the method for the sample selection.

As for the efficiency of sampling, the simulation results showed the MCM-8 improved sampling efficiency up to 1.7 times compared with SRS. To instantiate this in the context of our research, we selected 8 districts from 413 districts of Iran in the simulation. With each iteration in SRS, a completely different set of districts would be selected, varying from one to another and the resulting estimates were not stable. But with the proposed method, the selection was done between homogenous districts within a cluster. Therefore, the variability of sample estimates by the proposed sampling method was lower than the estimates made by SRS. The more homogeneous the cluster, the more efficient the sampling design [4, 5, 22]. This innovative way to define strata based on clustering methods is an efficient alternative to conventional stratified sampling. This property is particularly desired in surveys with small sample size, which are prone to a larger variability of sampling results.

Of note, the efficiency of our proposed sampling method is measured by DSO indicators as a proxy measures of targeted health conditions. These indicators only relatively specify the aspects of quality and cost. Therefore, steps should be taken to include as much as inclusive, relevant, and precise prior information of quality and costs of health conditions for sampling.

Disease-specific surveys such as IQCAMP require large registries and health information systems that are barely available in developing countries. Usually, the information on the resource use (cost and utilization) and quality of services of different health conditions are limited to small samples collected by non-representative sampling methods such as convenient sampling [26–28]. Thus, the proposed clustering method is very appealing for developing countries where healthcare data are limited. This strategy helps policymakers to conduct small sample size surveys with a limited budget.

The sampling unit is not restricted to hospital-based sampling and different types of primary sampling units can be selected in each stratum. In IQCAMP study, the primary sampling units were households for two conditions i.e., diabetes and road injuries, and hospital and outpatient clinics for the other six conditions. Therefore, the proposed design could be considered as a general design and can be used for any target population given that prior information about the outcome is available. Worth to note that, based on survey variable and the choice of proxy measures, the clustering results should be updated in the future studies. Thus, the overall sampling framework, not the clustering results are generalizable to the other settings.

The method works well if an expert team and technical modeling reinforce each other. We therefore recommend involving experts in variables selection and the evaluation of model results. The expert should also check the features of clusters that are identified through DTL. Otherwise, results would be less meaningful as clustering might be created based on variables that are found unimportant by experts. Furthermore, we recommend future research consider extending the method to creates clusters based on the importance of variables, for example, through a weighting system for variables.

The present study is subject to limitations. The first limitation regards the availability of district level data for some of the input measures. For district with no prior information, we used information available at their corresponding province. The second limitation refers to the representativeness of sampling results. The proposed method lies in the middle of a spectrum of sampling methods with convenient sampling methods at one extreme and SRS at the other. Though the sampling method is far away from convenient sampling, an extent to which it comes closer to a representative sampling is unclear and needs to be evaluated in the future studies. The third limitation refers to the external validity of this method, which needs to be examined in the future studies. The validity of the method is also linked to the appropriateness of the input indicators of studies. Using inclusive, relevant, and precise prior information of quality and costs of health conditions, the future studies could benefit from the efficiency of this stratified sampling design.

For the simplicity of sampling design, we used a common definition of strata for all eight health conditions in this research. This was motivated by the fact that access to prior information for each condition was limited. Furthermore, this common definition facilitated the administrative arrangement for data collection. However, with sufficient information per health condition, the definition of strata based on condition-specific outcomes could increase the sampling efficiency. We therefore call future research to address efficiency gain, cost, and feasibility of using condition-specific health outcomes to define strata for health conditions that are studied in the present research.

## Conclusions

The use of data mining approach improved the efficiency of sampling and markedly reduced the number of strata, i.e., geographical regions in the case study. The efficiency of proposed stratified sampling design was up to 1.7 times greater than SRS. Using this sampling design, the number of provinces that should be considered for sampling reduced from 31 to 8. Consequently, IQCAMP study deems nationally representative by only recruiting 300 participants per condition from the entire country. The proposed sampling design also identified key variables such as death from stroke, COPD, and in-hospital mortality that could be used as tracers to distinguish between districts in Iran for sampling from these target populations in the future studies.

## Supplementary Information

**Additional file 1.** Theories, methods, and results of model-based clustering. This file presents the theoretical aspects of model-based clustering method (part A). We also compared different model-based clustering methods (part B) and presented some results of model-based clustering at the provincial level (part C).

**Additional file 2.** R Code. This file includes R codes for all analyses that we have done in the manuscript. These analyses comprise the assessment of clustering tendency; determine the optimum number of clusters; conducting model-based clustering and hierarchical clustering method; internal validity; stability validity; identification of key features of clusters; and estimating sampling efficiency.

## Data Availability

The data that support the findings of this study are available from Non-Communicable Disease Research Center (NCDRC), Tehran, Iran. Data are available from the authors upon reasonable request and with permission of NCDRC.

## Abbreviations

APN: Average Proportion of Non-overlap
AD: Average Distance
ADM: Average Distance between Means
BIC: Bayesian Information Criteria
CKD: Chronic Kidney Disease
COPD: Chronic Obstructive Pulmonary Disease
DHS: Demographic Health Surveys
DM: Diabetes Mellitus
DRS: Death Registration System
DSO: Demand, Structure and Outcome
DTL: Decision Tree Learning
FOM: Figure of Merit
HCM: Hierarchical Clustering Method
IHD: Ischemic Heart Disease
IQCAMP: Iran Quality of Care in Medicine Program
MCM: Model-based Clustering Method
SBP: Systolic Blood Pressure
SRS: Simple Random Sampling
STEPS: STEPwise approach to Surveillance Non-Communicable Diseases study
