# Supplementary material for: Introducing an efficient sampling method for national surveys with limited sample sizes: application to a national study to determine quality and cost of healthcare
Source: BMC Public Health. 2021 Jul 17;21:1414. doi: 10.1186/s12889-021-11441-0 (PMC8285867; doi:10.1186/s12889-021-11441-0)
Supplement: Supplementary file 1 — Additional file 1. Theories, methods, and results of model-based clustering. This file presents the theoretical aspects of model-based clustering method (part A). We also compared different model-based clustering methods (part B) and presented some results of model-based clustering at the provincial level (part C). [file 12889_2021_11441_MOESM1_ESM.docx]

# Additional file 1

**Introducing an Efficient Sampling Method for National Surveys with Limited Sample Sizes: Application to a National Study to Determine Quality and Cost of Healthcare.**

## Part A: Theoretical Aspects of Model-based Method

In mathematical notation, imagine you have data on several attributes (a matrix X which includes several variables for different individuals) and you want to classify these individuals into G homogeneous cluster which suitably shows all the differences in the data set. We can consider X as coming from a mixture of densities as follows:

$$f\left( x \right)=\sum_{k=1}^{G} \tau_{k}f_{k}(x)$$

Where, fk is the probability density function of the observations in cluster k, and $\tau_{k}$ is the probability that an observation comes from the kth cluster ($\tau_{k}\in(0,1)$ and $\sum_{k=1}^{G} \tau_{k}=1$). Each cluster is usually modeled by the normal or Gaussian distribution. The distributions in each cluster are characterized by the mean $\mu_{k}$ and the covariance matrix $\Sigma_{k}$, which has the following probability density function:

$$\phi\left( x_{i};\mu_{k},\Sigma_{k} \right)=\frac{exp\left\{ -\frac{1}{2}\left( x_{i}-\mu_{k} \right)^{t}\Sigma_{k}^{-1}(x_{i}-\mu_{k}) \right\}}{\sqrt{det(2\pi\Sigma_{k})}}$$

There are a number of possible parameterizations of $\Sigma_{k}$**,** some of them are parameterized to impose constraints across components. In practice, we selected the best model specification based on model comparison criteria such as BIC. We have used R programming language using “mclust” package to estimate the best set of clusters accordingly.

## Part B: Comparison of Different Model-based Clustering Methods

Based on BIC model selection criteria, model-based clustering algorithm with 8 clusters and EEE (ellipsoidal, equal volume and shape) covariance specification for the multivariate normal distribution had the highest BIC value and this specification was selected for clustering the data (Additional file1-Figure 1).

Additional file1-Figure 1: Comparison of different model-based clustering based on BIC criteria.

| Note: Parameterizations of the covariance matrix $\Sigma_{k}$ available in mclust. | | | | |
| --- | --- | --- | --- | --- |
| Identifier | Distribution | Volume | Shape | Orientation |
| EII | Spherical | Equal | Equal | NA |
| VII | Spherical | Variable | Equal | NA |
| EEI | Diagonal | Equal | Equal | Coordinate axes |
| VEI | Diagonal | Variable | Equal | Coordinate axes |
| EVI | Diagonal | Equal | Variable | Coordinate axes |
| VVI | Diagonal | Variable | Variable | Coordinate axes |
| EEE | Ellipsoidal | Equal | Equal | Equal |
| EEV | Ellipsoidal | Equal | Equal | Variable |
| VEV | Ellipsoidal | Variable | Equal | Variable |
| VVV | Ellipsoidal | Variable | Variable | Variable |

## Part C: Clustering Results at Province Level

Since the input data is at the district level, MCM assigns districts into clusters. To generalize the clustering result at province level, we assigned province to a cluster that the majority of its districts (as also weighted by population size) fall into that cluster. To select one province from each cluster, we calculated the distance of each province from other provinces in the same cluster and selected the province with minimum distance from other provinces. This selection helps us to include a province which is a better representation of each cluster. The geographic distribution of MCM-8 at province level and the name of selected provinces are presented in Additional file1-Figure 2 and Additional file1-Table 1 respectively.

Additional file1-Figure 2: Geographic distribution of MCM-8 at the province level

Note: Markazi(MK), Gilan(GI), Mazandaran(MN), Azerbaijan-East(EA), Azerbaijan-West(WA), Kermanshah (BK), Khuzestan(KZ), Fars(FA), Kerman(KE), Khorasan-Razavi(KR), Isfahan(ES), Sistan-and-Baluchestan(SB), Kurdistan(KD), Hamedan(HD), Chahar-Mahal-Bakhtiari(CM), Lorestan(LO), Ilam(IL), Kohkiluyeh-and-Boyer-Ahmad(KB), BS(Bushehr), Zanjan(ZA), Semnan(SM), Yazd(YA), Hormozgan(HG), Tehran(TE), AR(Ardebil), Qom(QM), Qazvin(QZ), Golestan(GO), Khorasan -North(KN), Khorasan-South(KS), Alborz(AL)

Additional file1- Table 1: List of selected provinces by the proposed sampling method

| Cluster number | List of provinces within each cluster | Name of selected province |
| --- | --- | --- |
| 1 | Markazi, Qazvin, and Hormozgan | Qazvin |
| 2 | Gilan, Mazandaran, Kermanshah, Isfahan, Hamedan, Alborz | Gilan |
| 3 | Azerbaijan-East, Fars | Fars |
| 4 | Azerbaijan-West, Kerman, Sistan-and-Baluchestan | Azerbaijan-West |
| 5 | Khuzestan, Golestan | Khuzestan |
| 6 | Tehran, Khorasan-Razavi | Tehran |
| 7 | Kurdistan, Lorestan, Zanjan, Ardebil, Qom, Khorasan -North | Lorestan |
| 8 | Chahar-Mahal-Bakhtiari, Ilam, Kohkiluyeh-and-Boyer-Ahmad, Bushehr, Semnan, Yazd, Khorasan-South | Khorasan-South |
