# Supplementary material for: Introducing an efficient sampling method for national surveys with limited sample sizes: application to a national study to determine quality and cost of healthcare
Source: BMC Public Health. 2021 Jul 17;21:1414. doi: 10.1186/s12889-021-11441-0 (PMC8285867; doi:10.1186/s12889-021-11441-0)
Supplement: Supplementary file 2 — Additional file 2. R Code. This file includes R codes for all analyses that we have done in the manuscript. These analyses comprise the assessment of clustering tendency; determine the optimum number of clusters; conducting model-based clustering and hierarchical clustering method; internal validity; stability validity; identification of key features of clusters; and estimating sampling efficiency. [file 12889_2021_11441_MOESM2_ESM.pdf]

```
# Required Libraries
------------------------------------------------------
#
remove.packages(c("clValid","maptools","rgeos","rgdal","spdep","mclust
"))
# packageUrl<- "https://cran.r-project.org/src/contrib/Archive/
devtools/devtools_1.12.0.tar.gz"
# install.packages(packageUrl, repos=NULL, type='source')
# library("devtools")
# install_version("clValid", version = "0.6-4", repos = "http://
cran.us.r-project.org")
# install_version("maptools", version = "0.8-40", repos = "http://
cran.us.r-project.org")
# packageUrl<- "https://cran.r-project.org/src/contrib/Archive/rgeos/
rgeos_0.3-15.tar.gz"
# install.packages(packageUrl, repos=NULL, type='source')
# install.packages("rgeos")
# install.packages("rgdal")
# install_version("spdep", version = "0.6-8", repos = "http://
cran.us.r-project.org")
# install_version("mclust", version = "5.2", repos = "http://
cran.us.r-project.org")
# install_version("RColorBrewer", version = "1.0-5", repos = "http://
cran.us.r-project.org")
# install_version("car", version = "2.1-2", repos = "http://cran.us.r-
project.org")
libs <-
c("rpart","rpart.plot","clValid","readstata13","foreign","mclust",
"maptools", "rgeos",
          "rgdal","spdep", "car", "RColorBrewer", "factoextra",
"NbClust", "clustertend", "fpc",
          "randomForest", "caret", "tidyverse", "gdata")
lapply(libs, require, character.only = TRUE)

# Input data
--------------------------------------------------------------
rm(list=ls())
setwd("C:/Users/MP/Desktop/_IQCAMP Sampling Article/Statistical
Analysis/Data")
PCA.data <- read.dta13("Input Data.dta")
shape31<-readShapePoly("Shape 31/omega.shp",IDvar ="SP_ID")
shape397<-readShapePoly("Final 397 shape/shape397.shp",IDvar ="SP_ID")
w.data <- read.dta("Population Data.dta")
colnames(PCA.data[,c(26,9,10,19,24)])
PCA.data<-PCA.data[,-c(26,9,10,19,24)]
### Missing values
PCA.data[is.na(PCA.data$SBP),"SBP"] = median(PCA.data$SBP, na.rm =
TRUE)
PCA.data[is.na(PCA.data$Cholesterol),"Cholesterol"] =
median(PCA.data$Cholesterol, na.rm = TRUE)
```

```
PCA.data[is.na(PCA.data$Glucose),"Glucose"] = median(PCA.data$Glucose,
na.rm = TRUE)

# Remove Outlier Data
-----------------------------------------------------
x <- PCA.data[,-c(1:4)]
x<- as.matrix(x)
# boxplot(x)
removeOutliers <- function(x,...){
  qnt <- quantile(x, probs = c(0.25,0.75) , na.rm =T)
  H <- 1.5*(qnt[2]-qnt[1])
  y <- x
  y[x < qnt[1]-H] <- qnt[1]-H
  y[y > qnt[2]+H] <- qnt[2]+H
  x <- y
}
newX <- apply(x,2,removeOutliers)
maxs <- apply(newX,2,max)
mins <- apply(newX,2,min)
newX <- as.data.frame(newX)
rownames(newX)<- rownames(PCA.data)
PCA.data[,-c(1:4)]<- scale(newX,scale = maxs-mins, center = mins)

        # Assessment of Clustering Tendency
---------------------------------------
        res <- get_clust_tendency(PCA.data[,-c(1:4)], n =
nrow(PCA.data)-1, graph = FALSE)
        res$hopkins_stat
        pdf(paste0(getwd(),"/Get Cluster Tendency.pdf"), width=6,
height=6)
        get_clust_tendency(PCA.data[,-c(1:4)], n = nrow(PCA.data)-1,
                   gradient = list(low = "steelblue",  high =
"white"))
        dev.off()

        # Optimum Number of Clusters
----------------------------------------------
        ### All 30 indecs
        nb <- NbClust(PCA.data[,-c(1:4)], distance = "euclidean",
                 min.nc = 2, max.nc = 15,
                 method = "complete", index ="all")
        pdf(paste0(getwd(),"/Optimal number of clusters.pdf"),
width=8, height=6)
        factoextra::fviz_nbclust(nb) + theme_minimal()
        dev.off()

# Rum Model-Based Clustering Method (MCM)
---------------------------------
(MB.cluster <- Mclust(PCA.data[,-c(1:4)]))
summary(MB.cluster)
```

```
cl.MB<-as.vector(MB.cluster$classification)
plot(MB.cluster, PCA.data[,-c(1:2)], what = "BIC")

# Hierearchical Clustering Method (HCM)
-----------------------------------
H.cluster <- hclust(dist(PCA.data[,-c(1:4)]), "complete")
# plot(H.cluster)
cl.H.2<-as.vector(cutree(H.cluster, 2))
cl.H.8<-as.vector(cutree(H.cluster, 8))
IQCAMP.data<-cbind(PCA.data,cl.MB,cl.H.2,cl.H.8)

    # Compare Clustering Algorithm: Internal Validity
-------------------------
    ### INternal Validity
    sapply(list(mb = MB.cluster$classification, hc2 =cl.H.2, hc8
=cl.H.8),
           function(c) cluster.stats(dist(PCA.data[,-c(1:4)]), c)
[c("within.cluster.ss","avg.silwidth", "dunn")])

    ### External Validity
    clmethods <- c("hierarchical","model")
    stab <- clValid(PCA.data[,-c(1:4)], nClust = 2:31, clMethods =
clmethods,
                    validation = "stability")
    # Display only optimal Scores
    optimalScores(stab)
    summary(stab)
    # plot(stab)

######################################################################
##############
############
####
########         Identification of Distinct Features of Clusters
########
####
############
######################################################################
##############
newX$cl.MB<-cl.MB
dtm<-
rpart(cl.MB~Inpatient+Outpatient+Inpatient51+Outpatient51+patientexcha
ngerate+

Complementary_insurance+Bed_density+SBP+Glucose+Cholesterol+pod_stroke
+

pod_copd+pod_ckd+pod_diabetes+Neonatal_mortality+Adverse_effect+
          Mortality_ratio+mortalityhospital,data=newX, method="class")
```

```
varImp(dtm)
rfm<-
randomForest(cl.MB~Inpatient+Outpatient+Inpatient51+Outpatient51+patie
ntexchangerate+

Complementary_insurance+Bed_density+SBP+Glucose+Cholesterol+pod_stroke
+

pod_copd+pod_ckd+pod_diabetes+Neonatal_mortality+Adverse_effect+
                    Mortality_ratio+mortalityhospital,data=newX )

importance(rfm)
varImpPlot(rfm)

pdf(paste0("DT graph.pdf"), height=4, width=8)
rpart.plot(dtm,box.palette = 0, type=3, extra=2)
dev.off()
p<-predict(dtm, newX, type="class")
table(IQCAMP.data$cl.MB, p)
mean(IQCAMP.data$cl.MB==p)

######################################################################
##############
############
####
########               Sampling Efficiency for pod_stroke
########
####
############
######################################################################
##############
newX$province<-PCA.data$province
newX$district<-PCA.data$district
newXweight<- merge(newX, w.data ,by=c("district","province"))
var.names<-c("Inpatient", "Outpatient", "Inpatient51", "Outpatient51",
"patientexchangerate",
             "SBP", "Glucose","Cholesterol",
"Complementary_insurance", "Bed_density",
             "pod_stroke", "pod_copd", "pod_diabetes", "pod_ckd",
"Neonatal_mortality",
             "Adverse_effect", "Mortality_ratio","mortalityhospital",
"pop")
set.seed(12345); niter=1000; nclust=8
sim.srs  <-matrix(NA, nrow = niter, ncol = length(var.names)-1)
sim.clust<-matrix(NA, nrow = niter, ncol = length(var.names)-1)

for (i in 1:niter){
  ### SRS Sampling
  no.srs<-sample(1:dim(newXweight)[1], nclust)
  out.srs<-newXweight[no.srs,var.names]
```

```
  sim.srs[i,]<-as.numeric(lapply(X = out.srs[,c(1:18)], 2,FUN
=weighted.mean,w=out.srs[,c(19)]))

  # Sampling based-on MB-cluster
  out.clust<-matrix(NA, nrow = nclust, ncol = length(var.names))
  for (j in 1:nclust){
    dt.cl<-newXweight[which(newXweight$cl.MB==j),var.names]
    s<-sample(1:dim(dt.cl)[1],1)
    out.clust[j,]<-as.numeric(dt.cl[s,])
  }
  sim.clust[i,]<-as.numeric(lapply(X =
data.frame(out.clust[,c(1:18)]), 2,FUN
=weighted.mean,w=out.clust[,c(19)]))
}
sd.srs<-sd.clust<-eff<-c()
for (j in 1:(length(var.names)-1))
{
  sd.srs[j]<-sd(sim.srs[,j])
  sd.clust[j]<-sd(sim.clust[,j])
  eff[j]<-var(sim.srs[,j])/var(sim.clust[,j])
}
(cbind(var.names[-19], round(sd.srs, digits=3), round(sd.clust,
digits=3), round(eff, digits = 1)))[c(5,11,12,14, 18,16, 17),]
(cbind(var.names[-19], round(sd.srs, digits=3), round(sd.clust,
digits=3), round(eff, digits = 1)))


# Clusters according to Provinces
-----------------------------------------
ProvinceClusters <-
matrix(0,length(levels(factor(IQCAMP.data$province))),3)
for(i in 1:length(levels(factor(IQCAMP.data$province))))
{
  IthProvince <- subset(IQCAMP.data,province == i-1 )
  IthProvince.mb <- IthProvince$cl.MB
  NUMBER.mb <- order(as.matrix(table(IthProvince.mb)),decreasing =
TRUE)[1]
  ProvinceClusters[i,3] <-
strtoi(rownames(as.matrix(table(IthProvince.mb)))[NUMBER.mb])
  ProvinceClusters[i,1] <- IthProvince$province[1]
  ProvinceClusters[i,2] <- as.character(IthProvince$PROVINCE[1])
}
colnames(ProvinceClusters)<-c("PROVINCEID","PROVINCE", "cluster.mb" )
ProvinceClusters <- data.frame(ProvinceClusters)
#View(ProvinceClusters)
IQCAMP.data$cl.MB <- as.factor(IQCAMP.data$cl.MB)

    # Province Distance  Weighted
--------------------------------------------
    merge.data <- merge(IQCAMP.data,
```

```
w.data ,by=c("district","province"))
    table(is.na(merge.data$pop))
    ProvinceDistanceMatrix <- matrix(0,31,31)
    for(i in 0:29)
      for(j in i+1:29)
        if(j <= 30 & i!=25 & j!=25)
        {
          s1 <- subset(merge.data, province == i)
          s1 <- as.matrix(s1[,c((5:22),26)])
          sma1 <- apply(s1[,1:18], 2, FUN=weighted.mean,weights=s1[,
19])
          s2 <- subset(merge.data, province == j)
          s2 <- as.matrix(s2[,c((5:22),26)])
          sma2 <- apply(s2[,1:18], 2,FUN=weighted.mean, weights=s2[,
19])
          dis = (sma1-sma2)
          dis = dis^2
          ProvinceDistanceMatrix[i+1,j+1] <- sum(dis)
        }
    ####################################
    for (i in 25:25) ###Qom weight is 1
      for (j in i+1:29)
        if(j <= 30)
        {
          s1 <- subset(merge.data, province == i)
          s1 <- as.matrix(s1[,c(5:22)])
          sma1 <- apply(s1 , 2 , sum)
          s2 <- subset(merge.data, province == j)
          s2 <- as.matrix(s2[,c((5:22),26)])
          sma2 <- apply(s2[,1:18], 2,FUN=weighted.mean,weights=s2[,
19])
          dis = (sma1-sma2)
          dis = dis^2
          ProvinceDistanceMatrix[i+1,j+1] <- sum(dis)
        }

    for (i in 0:24 ) ###Qom weight is 1
      for (j in 25:25)
      {
        s1 <- subset(merge.data, province == i)
        s1 <- as.matrix(s1[,c((5:22),26)])
        sm1 <- apply(s1[,1:18], 2,FUN=weighted.mean , weights=s1[,19])
        s2 <- subset(merge.data, province == j)
        s2 <- as.matrix(s2[,c(5:22)])
        sma2 <- apply(s2 , 2 , sum)
        dis = (sma1-sma2)
        dis = dis^2
        ProvinceDistanceMatrix[i+1,j+1] <- sum(dis)
      }
```

```
### Select Provinces from MCM Clusters
###Based on 8 cluster
dis.data<-ProvinceDistanceMatrix
select.pro <- rep(NA,8)
for (c in 1:8)
  {
  pro.clust <-
sort(unique(IQCAMP.data$province[IQCAMP.data$cl.MB==c ]))
  pro.clust <- pro.clust+1
  nrow(dis.data)
  ncol(dis.data)
  x <- matrix(0 ,length(pro.clust) , length(pro.clust) )
  colnames(x) <- pro.clust
  rownames(x) <- pro.clust
  for (i in 1 : length(pro.clust))
    for (j in 1 : length(pro.clust))
      #if (j <= length(pro.clust))
    {
      {
        x[ i , j] = dis.data[(pro.clust[i]),(pro.clust[j])]
      }
    }
  lowerTriangle(x,diag=F, byrow=T) = upperTriangle(x,diag=F)
  #diag(x) = NA
  mean.pro <- apply(x , 2 , mean , na.rm=T)

  select.pro[c] <- names(which.min(mean.pro))  ######based on 1 to
31 province codes
}

#############final result
######based on 1 to 31 province codes
select.pro

# Plots at Provincial levels
----------------------------------------------
data.shape31 = attr(shape31, "data")
districtname<-c("MK","GI","MN","EA","WA","BK","KZ","FA",
            "KE","KR","ES","SB","KD","HD","CM","LO","IL",
            "KB","BS","ZA","SM","YA","HG","TE","AR","QM",
            "QZ","GO","KN","KS","AL")
order <- match(data.shape31$SP_ID, ProvinceClusters$PROVINCEID  )
order
ProvinceClusters<-ProvinceClusters[order,]
ProvinceClusters$District<-districtname
rownames(ProvinceClusters)<-ProvinceClusters$PROVINCEID
xx.profile <- spCbind(shape31,ProvinceClusters)
data.shape31 = attr(xx.profile, "data")

###   Model based clustering
```

```
    col <- rev(brewer.pal(8,"Spectral"))
    regionnames <- c("Cluster1", "Cluster2", "Cluster3", "Cluster4",
                    "Cluster5", "Cluster6", "Cluster7", "Cluster8")

    # Create list object for sp.layout
    sp.label <- function(x, label) {list("sp.text", coordinates(x),
label)}
    NUMB.sp.label <- function(x) {sp.label(x,
as.vector(x@data$District))}
    make.NUMB.sp.label <- function(x) {do.call("list",
NUMB.sp.label(x))}

    # Spplot
    # tps <- list(fontsize=list(text=5), fontcolor=list(text="green"))
    # trellis.par.set(tps)

    pdf(paste0("Province-level results_Model based clustering
method.pdf"), height=8, width=8)
    par(mfrow=c(1,1), mar=c(4,4,4,4))
    spplot(xx.profile, "cluster.mb", col.regions =col,
col="gray30",cex.main=3,
           main=paste("Clustering of Prior Information",
                      "using Model-based Method (MCM)",sep="\n"),
            colorkey = list(labels = list( labels =regionnames),width
= 1, cex = 5),
           sp.layout = make.NUMB.sp.label(xx.profile), cex.main=1.5)
    dev.off()

    # Plots at District levels
----------------------------------------------
    data.shape397 = attr(shape397, "data")
    order <- match(data.shape397$SP_ID,IQCAMP.data$district)
    order
    IQCAMP.data<-IQCAMP.data[order,]
    rownames(IQCAMP.data)<-IQCAMP.data$district
    xx.profile <- spCbind(shape397,IQCAMP.data)
    table(xx.profile$cl.MB)

    ###   Model based clustering
    pdf(paste0("District-level results_Model based clustering
method.pdf"), height=6, width=8)
    par(mfrow=c(1,1), mar=c(4,4,4,4))
    col <- rev(brewer.pal(8,"Spectral"))
    regionnames <- c("Cluster1: 31 districts", "Cluster2: 86
districs", "Cluster3: 49 districts", "Cluster4: 59 districts",
                    "Cluster5: 40 districts", "Cluster6: 42
districts", "Cluster7: 45 districts", "Cluster8: 61 districts")

    spplot(xx.profile, "cl.MB", col.regions =col, col="gray30",
           cex.main=3, main=paste("Clustering of Prior Information at
```

```
District Level",
                                  "using Model-based Method
(MCM)",sep="\n"),
           colorkey = list(labels = list( labels =regionnames),width =
2, cex = 5))
    dev.off()
```
